# 14. Effects of an Opt-Out Protocol for Antibiotic De-escalation among Selected Patients with Suspected Sepsis: The DETOURS Trial

**DOI:** 10.1093/ofid/ofab466.014

**Published:** 2021-12-04

**Authors:** Rebekah W Moehring, Michael E Yarrington, Bobby G Warren, Yuliya Lokhnygina, Erica Atkinson, Allison Bankston, Julia Coluccio, Michael Z David, Angelina Davis, Janice Davis, Brandon Dionne, April Dyer, Travis M Jones, Michael Klompas, David W Kubiak, John Marsalis, Jacqueline Omorogbe, Patricia Orajaka, Alice Parish, Todd Parker, Jeffrey C Pearson, Tonya Pearson, Christina Sarubbi, Christian Shaw, Justin Spivey, Robert Wolf, Rebekah Wrenn, Elizabeth Dodds Ashley, Deverick J Anderson

**Affiliations:** 1 Duke Center for Antimicrobial Stewardship and Infection Prevention, Durham, NC; 2 Duke University School of Medicine, Durham, North Carolina; 3 Southeastern Regional Medical Center, Lumberton, North Carolina; 4 Piedmont Newnan Hospital, Newnan, Georgia; 5 Piedmont Healthcare, Atlanta, GA; 6 University of Pennsylvania, Philadelphia, Pennsylvania; 7 Piedmont Fayette Hospital, Fayette, Georgia; 8 Brigham and Women’s Hospital, Boston, Massachusetts; 9 Harvard Medical School and Harvard Pilgrim Health Care Institute, Boston, Massachusetts; 10 Brigham and Women's Hospital, Boston, Massachusetts; 11 Iredell Health, Statesville, North Carolina; 12 Duke University, Durham, North Carolina; 13 UNC REX Healthcare, Raleigh, North Carolina; 14 Wilson Medical Center, Wilson, North Carolina; 15 Duke University Medical Center, Durham, North Carolina; 16 Boston University School of Medicine, Boston, California

## Abstract

**Background:**

Sepsis guidelines recommend daily review to de-escalate or stop antibiotics in appropriate patients. We conducted a randomized controlled trial (NCT03517007) of an opt-out protocol to decrease unnecessary antibiotics in selected patients with suspected sepsis.

**Methods:**

We evaluated non-ICU adults remaining on broad-spectrum antibiotics with negative blood cultures at 48-96 hours at ten U.S. hospitals during September 2018-May 2020. A 23-item safety check excluded patients with ongoing signs of infection, concerning or inadequate microbiologic data, or high-risk conditions (Figure 1). Eligible patients were randomized to the opt-out protocol vs. usual care. The primary outcome was 30-day post-enrollment antibacterial days of therapy (DOT). Clinicians caring for intervention patients were contacted by a pharmacist or physician to encourage antibiotic discontinuation or de-escalation using opt-out language, discuss rationale for continuing antibiotics, working diagnosis, and de-escalation and duration plans. Hurdle models separately compared the odds of antibiotic continuation and DOT distributions among those who continued antibiotics.

Components of the De-Escalating Empiric Therapy: Opting-OUt of Rx in Selected patients with Suspected Sepsis (DETOURS) Trial Protocol

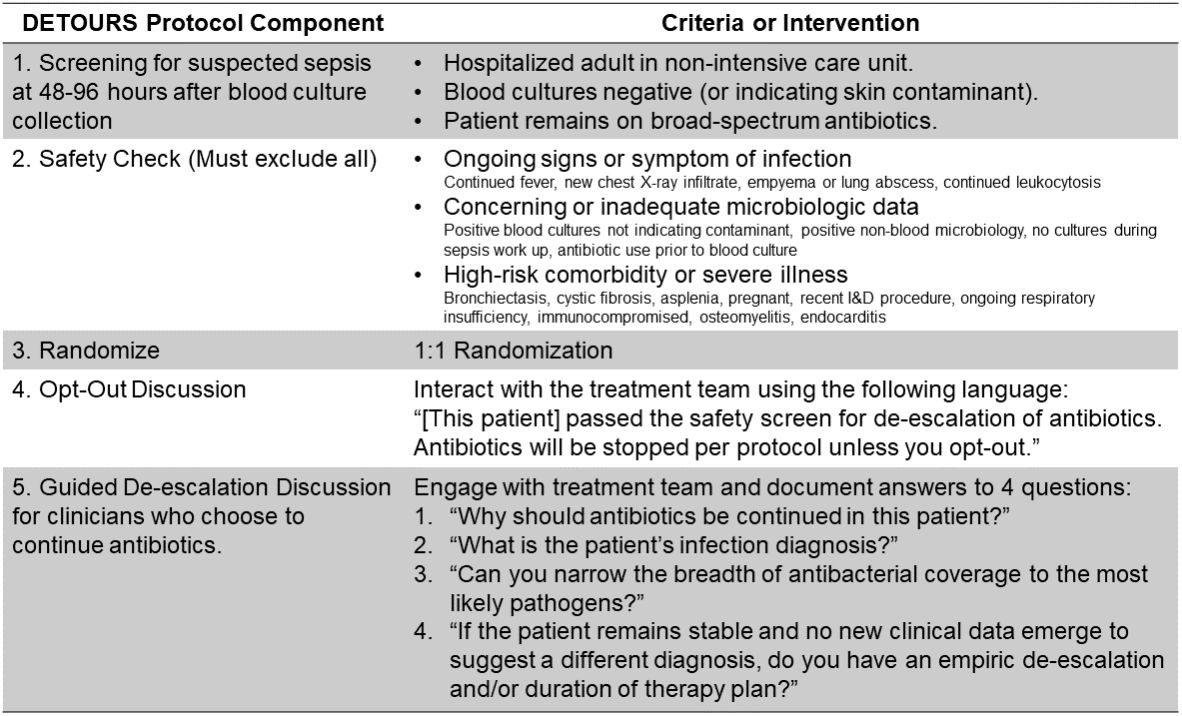

**Results:**

Among 9606 screened, 767 (8%) were enrolled (Figure 2). Common reasons for exclusion were antibiotics given prior to blood culture (35%), positive culture from non-blood sites (26%), and increased oxygen requirement (21%). Intervention patients had 32% lower odds of antibiotic continuation (79% vs. 84%, OR 0.68, 95% confidence interval [0.47, 0.98]). DOT distributions among those who continued antibiotics were similar (ratio of means 1.06 [0.88-1.26], Figure 3). Fewer intervention patients were exposed to extended-spectrum agents (38% vs. 44%). Common reasons for continuing antibiotics were treatment of localized infection (76%) and belief that stopping antibiotics was not safe (31%). Safety outcomes such as mortality, readmission, sepsis relapse, *C. difficile*, and length of stay did not differ.

DETOURS Trial Flow Diagram

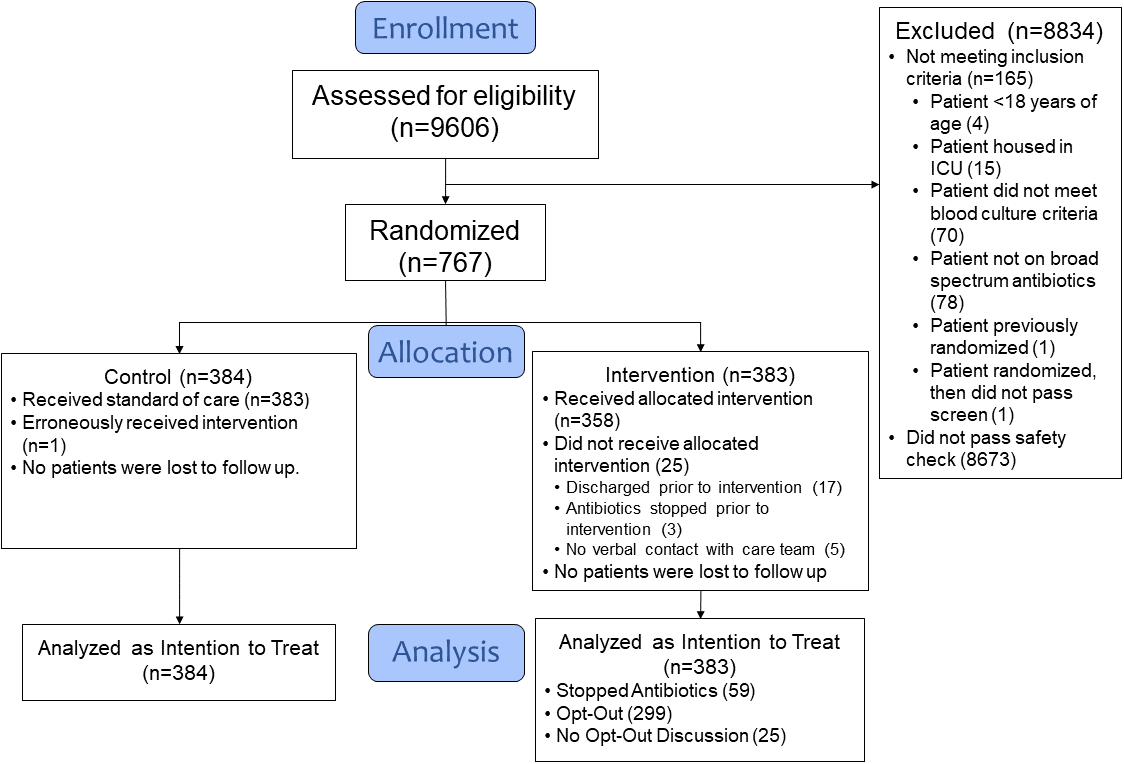

Flow of participants through the DETOURS Trial.

Observed Days of Antibiotic Therapy Among Intervention and Control Subjects in the DETOURS Trial

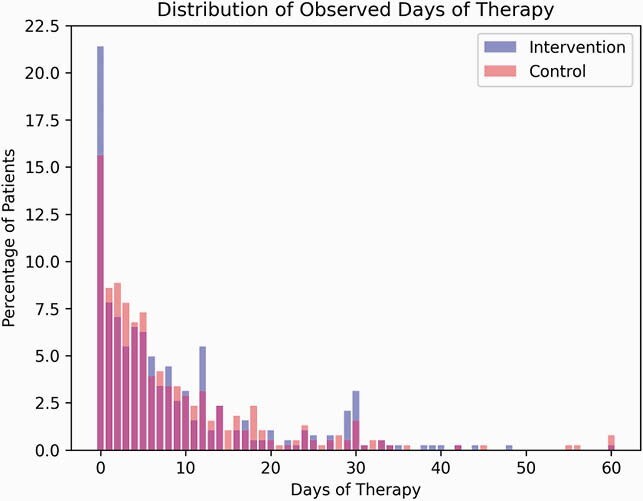

Post-enrollment days of antibiotic therapy among 767 DETOURS Trial participants in 10 US acute care hospitals within 30 days after enrollment. Dark pink color indicates percent overlap between intervention (purple) and control (light pink) groups.

**Conclusion:**

In this patient-level randomized trial of a stewardship intervention, the opt-out de-escalation protocol targeting selected patients with suspected sepsis resulted in more antibiotic discontinuations but did not affect safety events.

**Disclosures:**

**Rebekah W. Moehring, MD, MPH**, **UpToDate, Inc.** (Other Financial or Material Support, Author Royalties) **Michael Z. David, MD PhD**, **GSK** (Board Member) **Michael Klompas, MD, MPH**, **UpToDate** (Other Financial or Material Support, Chapter Author)

